# Oral Tablet Formulations with Lactoferrin, a Cohesive Biomacromolecule

**DOI:** 10.3390/pharmaceutics17091151

**Published:** 2025-09-02

**Authors:** True L. Rogers, Andrew J. Horton, Thomas Watson, Stephanie Robart, Brooklynn DeFrancesco, Hannah Bishop, Elizabeth Tocce

**Affiliations:** Roquette, Midland, MI 48642, USA

**Keywords:** lactoferrin, protein, biologic, cohesive API, oral solid dosage form, hydroxypropyl methylcellulose, microcrystalline cellulose, croscarmellose, lactose, stability

## Abstract

**Background/Objectives:** The aim of our research was to understand how excipients, unit operations, and process parameters impact processability and resulting properties, performance, and stability of tablets containing bovine lactoferrin, a cohesive biomacromolecule. **Methods:** Microcrystalline cellulose (MCC), croscarmellose (xCMC), lactose (LAC), hydroxypropyl methylcellulose (HPMC), and sodium stearyl fumarate (SSF) were used to produce various tablet formulations containing lactoferrin across a concentration range of 5 to 45%, targeting immediate- or controlled release performance. Tablets were made either by direct compression or via dry granulation followed by tableting. In addition to release performance, tablet attributes were characterized for tensile strength, friability, weight uniformity, and content uniformity. **Results:** Acceptable tablet tensile strength, friability, and performance were obtained for lactoferrin concentrations ranging from 15 to 45%, using a variety of excipients and manufacturing approaches. In several cases, dry granulation improved content uniformity. Excipient choice and tablet compression force impacted drug release, particularly when MCC alone was used as dry binder for immediate release. Dry granulation impacted tablet tensile properties, but did not significantly impact release performance. Lactoferrin–excipient compatibility was demonstrated for up to 2 years in ambient laboratory conditions. **Conclusions:** The study demonstrates that robust tablets can be produced using excipients and processes amenable to scale-up for industrial production. Consistent, stable, and suitably performing tablets were successfully produced using a variety of excipients, processing approaches, and across a broad concentration range with this cohesive biomacromolecule active pharmaceutical ingredient (API). Both immediate- and controlled release performance modes were possible.

## 1. Introduction

Oral delivery of peptides and proteins has been an alluring area of research since 1922, when insulin was first isolated [1]. There are over 1000 active programs for orally delivered biomacromolecules to treat various disease states, including infection, obesity, diabetes, autoimmune diseases, and digestive enzyme deficiency, with multiple product approvals [2]. That stated, biomacromolecules are mainly administered via injection, but patients typically prefer oral dosage forms over injectables due to accessibility, convenience, and an injection-free experience [3]. Studies of Novo-Nordisk’s *Rybelsus* oral caplets containing the modified glucagon-like peptide (GLP-1) agonist, semaglutide, suggest the oral formulation is an effective alternative to the subcutaneous injectable formulation [4], albeit requiring higher dosages. The Rybelsus caplet formulation contains a high concentration of the absorption enhancer salcaprozate sodium, which enables about 1% oral absorption of semaglutide [5]. Hence, there are fundamental challenges in oral biomacromolecule formulation and delivery, the most notable being the need to improve oral bioavailability [6,7,8,9,10,11]. Other challenging but less discussed issues include (1) accommodating the cohesive and low-density properties of biomacromolecule powders isolated from freeze- or spray-drying [12,13,14], (2) attaining acceptable formulation processability and resulting dosage form properties [5,15], (3) delivering the necessary performance mode (e.g., immediate release, controlled release, etc.), and (4) retaining the active form of the biomacromolecule active pharmaceutical ingredient (API).

Cohesive API particles tend to adhere to each other, consequently negatively impacting formulation processability, as well as uniformity and quality of the resulting dosage form. There is ample literature on accommodating the cohesive properties of small-molecule APIs, such as acetaminophen, ibuprofen, and ketoprofen, via excipient and/or process selection [16,17,18,19,20,21].

There is limited literature for cohesive biomacromolecules [12,13,22], particularly regarding how excipient or process selection impacts production and performance of oral solid dosage forms (OSDF) containing these types of APIs. Hazlett et al. [12] reviewed agglomeration and coating processes to enhance flowability of high-protein dairy powders. Although the aim was for the food industry, the granulation/coating processes discussed are also relevant to the biopharmaceutics industry. Holmfred et al. [13] explored the compaction properties of binary mixtures containing either of two model proteins (lysozyme or bovine serum albumin [BSA]) and microcrystalline cellulose (MCC), spray-dried lactose (LAC) monohydrate, or calcium hydrogen phosphate dihydrate. Lysozyme, with *D*_50_ particle size < 15 µm, provided stronger tablets than bovine serum albumin (BSA), with *D*_50_ particle size > 225 µm, and MCC provided at least 3× greater tensile strength and nearly 2× elastic recovery following tablet compression vs. the two brittle-fracture excipients. Holmfred concluded that both the biomacromolecule and excipient attributes impact tablet properties, and that tablets containing biomacromolecules could be produced by following similar pharmaceutical principles as tablets containing small-molecule APIs. With the focus of their study on tablet strength and elastic recovery, powder cohesion issues, release performance, and protein stability were not investigated.

Wei et al. [22] compacted neat BSA and reported aggregation of the protein upon compression, highlighting the impact that processing could have on biomacromolecule structure; however, neither powder cohesion nor processability were investigated. Pedersen et al. [15] spray-dried insulin with maltitol, using starch as a proxy for a poorly compactible absorption enhancer, highlighting potential issues of formulating with this type of excipient. Their study focused on coprocessing insulin with maltitol to accommodate the processability challenges imparted by the absorption enhancer. Chen et al. utilized a wet-milling particle engineering technology to improve manufacturability of a cohesive and poorly compactable API for minitablet production, but biomacromolecules are typically formulated to OSDFs using low-moisture processes to minimize degradation of the active ingredient [13].

Chen [23], Vidakovic [24], Liu [25], Masloh [26], and Yang [27] et al. investigated various nanoparticulate or encapsulation technologies. Chen et al. produced β-glucan nanoparticles containing gemcitabine via film casting and cryo-milling for enhanced oral bioavailability. Vidakovic et al. produced lipidic calcein or insulin archaeosomes, microfluidizing to attain ~100 nm nanocapsules, which could then be isolated to powder via freeze- or spray-drying with minimal increase in redispersed nanocapsule size. They indicated that the archaeosome powders could be formulated to OSDFs, but downstream formulation was reserved for future studies. Liu, Masloh, and Yang et al. also investigated various nanoparticulate or encapsulation technologies, mainly for the purpose of improving bioavailability. These aforementioned nanoparticulate or encapsulation approaches could encounter processing challenges due to nanoparticulate size domains, particularly if they were isolated to powders via freeze- or spray-drying.

The aim of our research was to understand how excipients, unit operations, and process parameters impact processability and resulting properties, performance, and stability of tablets containing bovine lactoferrin, a cohesive model biomacromolecule, across an API concentration range of 5 to 45%. Particle morphology and powder density were investigated, as well as cohesive properties of both neat and blended powders. Both direct compression and roller compaction were assessed for tablet production as these are common processes for commercial OSDF manufacture. Wet granulation was not included in this study due to the tendency of biomacromolecules to degrade in the presence of moisture [13]. Tablet properties, such as weight, tensile strength, friability, and content uniformity, were investigated, and release performance was characterized using dissolution and disintegration testing. Intact lactoferrin content was also determined to gauge protein stability through formulation processing and tablet storage at ambient laboratory conditions.

## 2. Materials and Methods

### 2.1. Materials

Ingredients used to make the various tablet formulations are shown in Table 1. The materials were generally used as received, with the exception of lactose, which was sieved through a 20-mesh screen prior to use.

### 2.2. Powder Physical Properties

Particle size analysis was conducted using a Malvern Mastersizer 3000 equipped with an Aero S dry powder dispersion unit. Tapped density was measured using USP 47 <616> Method I (Measurement in a Graduated Cylinder, procedure I) [28] on a Vankel VanderKamp tester (Model 10700). A Scott Volumeter was utilized to measure bulk density of each material as specified in USP 47 <616> Method II (Measurement in a Volumeter). Scanning electron microscopy (SEM) was conducted using a Phenom Pro Desktop instrument (Nanoscience Instruments, Phoenix, AZ, USA) set at 10 kV and 200× magnification.

### 2.3. Schulze Ring Shear Testing

Powder was loosely filled and leveled into the small cell (TYPE S: 220 cm^3^) of an RST-01.pc ring shear tester (Dietmar Schulze). Fresh powder samples were measured at each of four normal stress levels (624, 2000, 4400, and 9400 Pa) using the ASTM method D6773 [29]. The yield loci at each stress level were generated using five points, which were determined at percentages of the maximum normal stress value. The data for the yield loci were acquired in duplicate at each percentage of the normal stress. Schulze software (RST-CONTROL 95 + RSV 95, Version 2.2.0.48, including RSV 95 version 3.x, Dietmar Schulze 2002–2019) was used to generate the flow functions. Flow functions of unconfined yield stress vs. maximum principle stress were plotted, along with reference lines to indicate different flow regimes, where increasing slope represented increasing powder cohesion.

### 2.4. Dry Granulation (Roller Compaction)

In addition to direct compression, each powder blend was also roller compacted and comilled before tableting. A model TF-Mini Vector Industrial roller compactor, equipped with a single-flight feed screw and DPS pressure rolls, was used for ribbon compaction. Roller compaction conditions were 5.4 rpm feed screw speed and 2.4 rpm roller speed. Ribbons were compacted at 700 lb (3.1 kN) of force. The compacted ribbons were passed through a Quadro Engineering CoMil (model 197S) using the round edge impeller and 2A-075R037/51 screen to obtain granules.

### 2.5. Tablet Formulations

All components except SSF were mixed in the V-blender for 10 min. For direct compression, SSF was then added with subsequent V-blending for one additional minute. For dry granulation, the blended powder was roller compacted, comilled, then lubricated for 1 min. See Table 2 for formulation details.

### 2.6. Tableting

The tablet formulations were compacted using the parameters listed in Table 3.

Tablets were equilibrated overnight at ambient conditions in sealed Ziploc bags prior to characterization.

### 2.7. Tablet Physical Attributes

Tablet weight, diameter, thickness, and breaking force were measured using a Sotax AT 50 instrument (Westborough, MA, USA). Tensile strength was calculated using Equation (1).

Equation (1): Tensile strength calculation.

(1) $$Tensile\;Strength=\frac{P\cdot 2}{\pi \cdot thickness\cdot diameter}$$

Tensile strength units are MPa or N/mm^2^, P is breaking force in Newtons (N), and tablet diameter and thickness units are mm.

### 2.8. Tablet Content Uniformity

For formulations containing 15, 30, or 45% lactoferrin, a tablet was weighed and transferred into a 500 mL volumetric flask. For the formulations containing 5% lactoferrin, a tablet was weighed and transferred into a 100 mL volumetric flask. The following steps were performed on all samples.

A magnetic stir bar was added into the flask, and pH 7.4 phosphate buffer was added up to the 500- or 100-mL volume marker. The flask was covered with parafilm, and the solution was stirred overnight alongside a flask of known lactoferrin concentration as a standard. This procedure was repeated in repetitions of six (n = 6) for each tablet formulation. After stirring overnight, the magnetic stir bar was removed, and the solution was poured into a 4 oz (120 mL) jar for sampling. Standard and sample solutions were passed through a 70 μm filter. Analysis was conducted on a Distek 2100 system equipped with an Agilent UV spectrophotometer (226 nm wavelength, 10 mm path length).

### 2.9. Drug Dissolution

The parameters listed in Table 4 were used to measure lactoferrin release from the tablet formulations.

Measurements were taken every 5 min for the first hour, then every 30 min to hour 5, and then every 60 min to hour 24. Data were not normalized.

The suspended hanging basket is a preferred methodology in our laboratories to minimize dissolution measurement error, and it was originally adapted using the compendial method for felodipine extended release (ER) tablets [30]. However, we use a basket that looks like a USP I basket, rather than the quadrangular basket described in the felodipine monograph.

*f*_2_ similarity factors were calculated using Equation (2) to compare release profiles [31]. *n* is number of time points; *R_t_* is percent API released from the reference product at time, *t*; and *T_t_* is percent API released from the test product at time, *t*. If *f*_2_ ≥ 50, then the release profiles are considered similar. If *f*_2_ = 100, then the release profiles are identical. If *f*_2_ < 50, then the release profiles are dissimilar.

Equation (2): *f*_2_ similarity factor equation.

(2) $$f_{2}=50\cdot log\left\{{\left[1+\frac{1}{n}\sum_{t=1}^{n}{\left(R_{t}-T_{t}\right)}^{2}\right]}^{-0.5}\cdot 100\right\}$$

### 2.10. Disintegration

Tablet disintegration was measured using a Sotax DT50 automated disintegration tester (Westborough, MA, USA). Eight hundred mL of pH 7.4 phosphate buffer was added into the beaker, the induction plate was allowed to sink to the bottom of the beaker, and the buffer was equilibrated to 37 °C. The bottom of each cell making up the sample holder held a 20 mm diameter screen made of plastic mesh with 2 mm openings. A single tablet was placed into each of the six cells of the sample holder. A detection disc was then placed in each of the six cells, with the disc detection height set at 0.5 mm. The sample holder was then attached to the reciprocating arm of the testing device and vertically oscillated in the buffer at 30 dips per min. Each tablet disintegration time was determined independently by each of the six detection discs. Once each tablet was no longer detected, that disintegration time was automatically marked for each tablet. The test did not conclude until all six tablets were no longer detected.

### 2.11. Enzyme-Linked Immunosorbent Assay (ELISA)

A bovine lactoferrin ELISA kit, ab274406, was purchased from Abcam (Waltham, MA, USA). Information for the bovine lactoferrin ELISA kit can be found at https://www.abcam.com/bovine-lactoferrin-elisa-kit-ab274406.html (accessed on 3 October 2023).

The ELISA methodology was very detailed and step-intensive, and the description is lengthy. It can be found in Appendix A.

### 2.12. Determination of Intact Lactoferrin Content in Tablet Formulations

pH 1.2 HCl + pepsin was prepared according to the United States Pharmacopeia recipe for simulated gastric fluid (SGF) [32]. pH 9.0 neutralizing buffer was prepared by dissolving 14.2 g sodium phosphate dibasic anhydrous in 1 L deionized water. Both buffers were warmed to 37 °C prior to use. For neutralization, 10 mL of pH 1.2 HCl + pepsin and 20 mL of pH 9.0 neutralizing buffer were added to a 50 mL centrifuge tube to attain a final pH of 7.

One IR tablet was added to each tube containing the neutralized media and vortexed for 1 min. Each neutralized sample was frozen until ELISA analysis was conducted. Each frozen neutralized IR tablet sample was removed from the freezer and rolled gently overnight on a roller situated in a refrigerator (4.8 °C) to thaw and fully dissipate. Controlled release HPMC matrix tablet formulations required longer rolling time to allow the matrix to fully dissipate and release the lactoferrin, so the HPMC matrix tablet samples were rolled for approximately 72 h at 4.8 °C.

The methodology of preparing the dissipated tablet samples for ELISA analysis was very detailed and step-intensive, and the description is lengthy. It can be found in Appendix B.

### 2.13. Stability

Tablets were stored in sealed Ziploc bags at ambient laboratory conditions. Temperature and humidity in this laboratory room were not monitored, but a neighboring laboratory room was monitored and experienced temperature and relative humidity over a two-year period of 21 ± 0.5 °C and 35 ± 10.9% RH, respectively.

## 3. Results

### 3.1. Powder Physical Attributes

**Figure 1:**
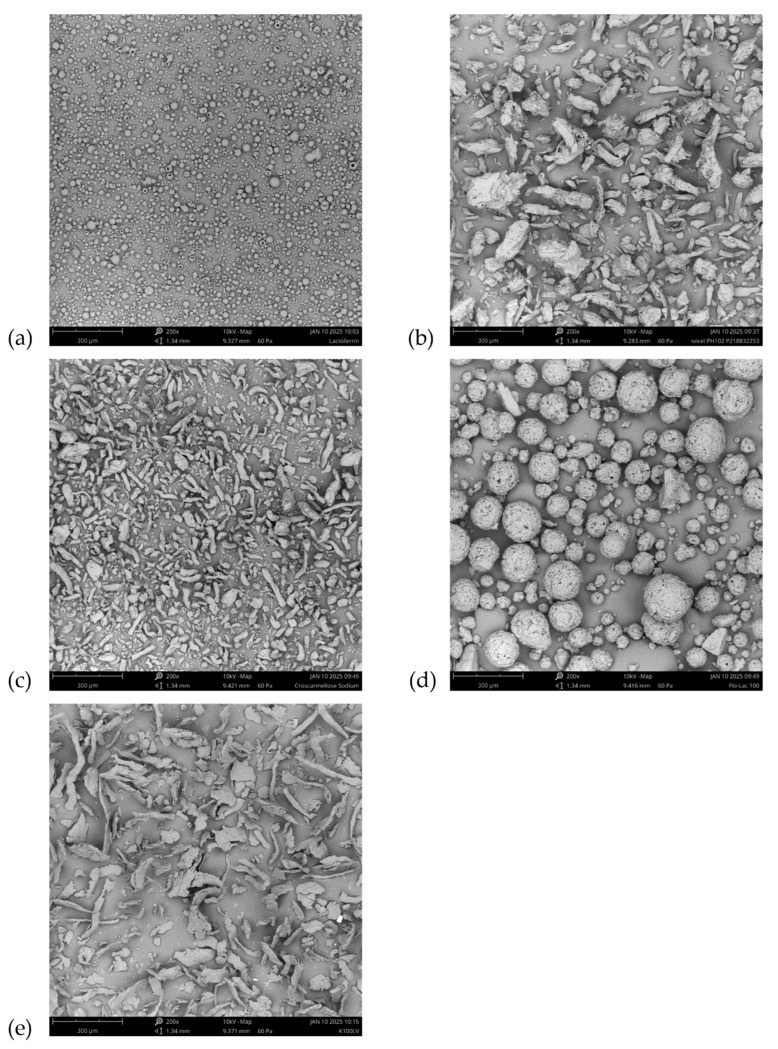
Scanning electron micrographs of (**a**) lactoferrin, (**b**) MCC, (**c**) xCMC, (**d**) LAC, and (**e**) HPMC. Magnification is 200×, and the scale bar is 300 µm. The 300 µm scale bar is in the lower left-hand corner of each SEM image.

The lactoferrin used in this study, supplied by Parchem, was likely isolated to powder by spray-drying, based upon its morphological appearance. SEM images of it and the excipients used to make the various tablet formulations are shown in Figure 1. Protein and peptide powders are typically isolated by spray-drying or freeze-drying, which can lead to morphological properties that present processability challenges during downstream unit operations, such as tableting.

It should be noted from its morphology (Figure 1d) that LAC also was isolated to powder via spray-drying, in this case resulting in a combination of fine and coarse spherical particles. This will be further discussed later. It should also be noted that, in the case of LAC, spray-drying resulted in a more flowable morphology, in contrast to the impact spray-drying had on producing the fine, cohesive morphology of lactoferrin.

#### 3.1.1. Bulk and Tapped Densities

If a formulation flows poorly during tableting, tablet weight variability will likely be high due to inconsistent die fill, and tablet weight may be below target due to incomplete die fill. If the formulation is poorly compactable, tablet tensile strength will likely be low, and friability will conversely increase. Typically, higher density corresponds to greater flowability [33] and lower compactability [34]. Compaction tendency will generally decrease as bulk and tapped densities converge (i.e., the Hausner ratio will be low), but flowability will increase. With a Hausner ratio of 1.22, LAC should exhibit poor compactability relative to the other ingredients (as shown in Table 5), but greater flowability. Lactoferrin and HPMC (with Hausner ratios of 1.65 and 1.80, respectively) indicate poor flowability and greater compactability. MCC (Hausner ratio 1.49) should be relatively balanced in flowability and compactability. Formulation HD/MCC, also with an intermediate Hausner ratio of 1.49, should be both flowable and compactable as well. Note: Only formulation HD/MCC was included in Table 5 for conciseness, since 45% lactoferrin concentration represents the high-dose upper boundary of the scoped study.

#### 3.1.2. Particle Size

**Figure 2:**
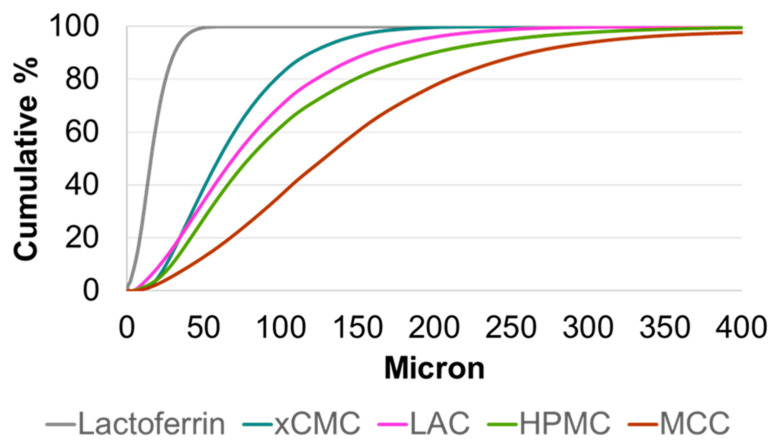
Malvern particle size distributions of lactoferrin and the excipients used to make the various tablet formulations.

Malvern particle size distributions (PSDs) of lactoferrin and the excipients are shown in Figure 2, and the *D*_10_, *D*_50_, *D*_90_, and *D*[4,3] particle size attributes are listed in Table 6. The lactoferrin sourced from Parchem is a fine powder with a narrow PSD. The particle size attributes of xCMC, LAC, and HPMC are comparable and coarser than that of lactoferrin. MCC was measured to be the coarsest of the ingredients.

Referring back to the SEM micrographs (Figure 1), one would have expected that the particle size attributes of LAC would have been more comparable to that of MCC, i.e., coarser than the Malvern measurement determined. Studying more closely the SEM micrograph for LAC (Figure 1d), a very broad distribution of particle size is observed in the image. There are many fine spherical particles, but also a large population of coarse spherical particles. This observation may explain why LAC was measured via Malvern to have particle size attributes closer to those of xCMC and HPMC. That stated, there is evidence of fine particles in the Malvern PSD profile for LAC in Figure 2, as its profile is broad, with more particles measured at the fine end of the PSD compared to those of xCMC and HPMC.

#### 3.1.3. Schulze Ring Shear

**Figure 3:**
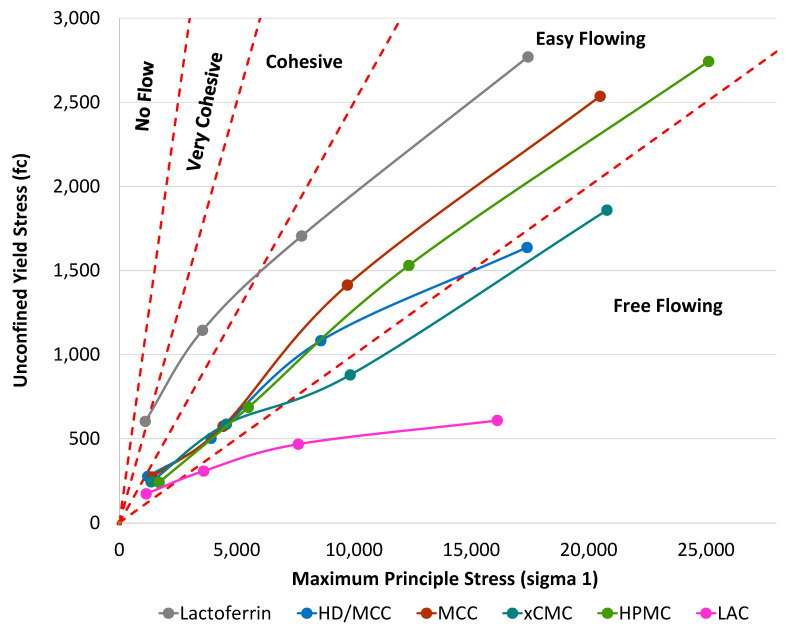
Unconfined yield stress vs. maximum principle stress profiles from ring shear testing. The red dashed reference lines indicate separations between the labeled flow regimes.

Powder cohesiveness can increase as particle size decreases [35], and this can result in processability challenges during formulation and tableting. Schulze ring shear data, shown in Figure 3, reveal the cohesive properties of lactoferrin and the excipients used in this study. Die fill during tableting would be considered a low stress environment, so particular attention is placed on ring shear data at low principal stress. MCC, xCMC, LAC, and HPMC had flow functions in the easy flowing regime at low stress, while the lactoferrin flow function was in the very cohesive regime.

**Figure A1:**
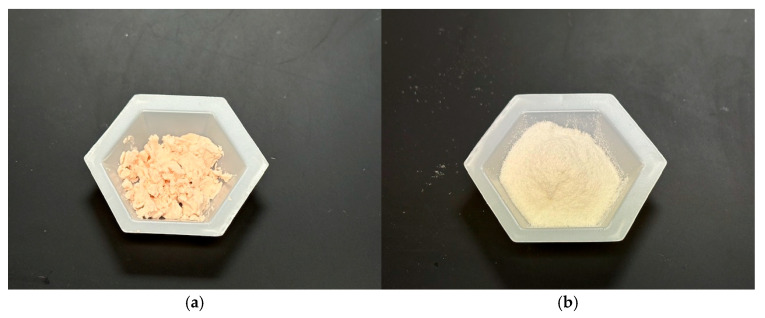

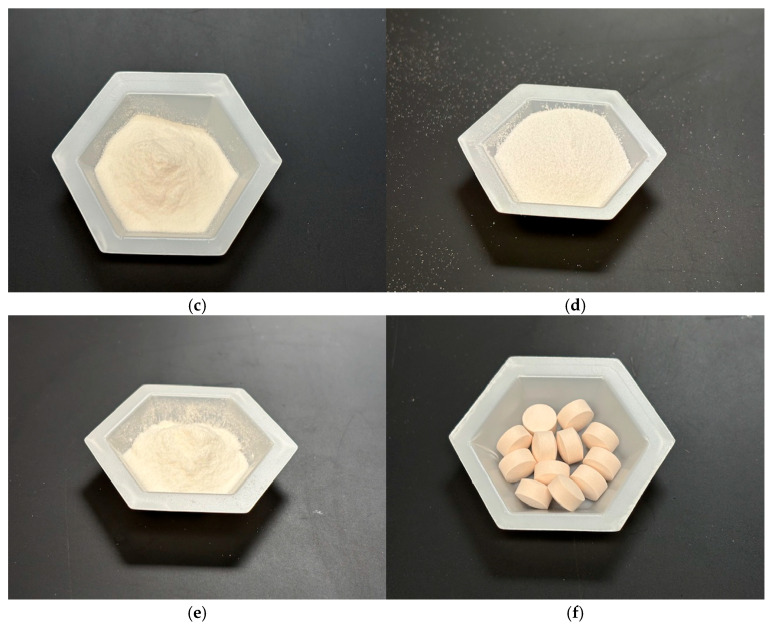
Macroscopic powder sample images of (**a**) lactoferrin, (**b**) MCC, (**c**) xCMC, (**d**) LAC, and (**e**) HPMC. (**f**) shows representative tablets produced in this study.

As previously described, lactoferrin is considered compactable but poorly flowing, with its fine particle size, low bulk density, and high Hausner ratio. Ring shear data corroborated the particle size and density attributes of lactoferrin, all characterizing it as cohesive to very cohesive powder that is poorly flowable and difficult to process. Furthermore, the macroscopic appearance of the powder confirms lactoferrin as a very cohesive material (see macroscopic powder image in Appendix C Figure A1).

Blending lactoferrin with MCC (HD/MCC) delivered a flow function on the border between cohesive and easy flowing at lowest stress, but then transitioning into the easy flowing regime. The ring shear data from the powder mixture corroborate the density and Hausner ratio data, implying that a direct compression powder blend containing 45% lactoferrin with MCC could be sufficiently processable. The remainder of the paper will be dedicated to determining how processable lactoferrin can be when combined with the excipients used in this study to produce the various tablet formulations.

### 3.2. Tablet Physical Attributes and Performance

#### 3.2.1. Tensile Strength

**Figure 4:**
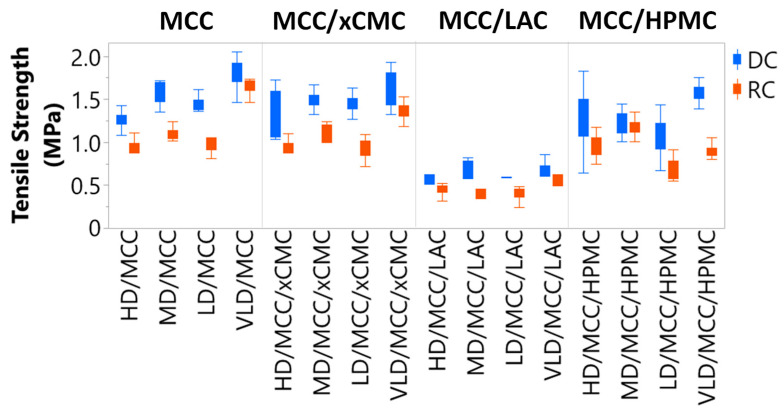
Tensile strengths of tablets produced at 2.2 kN compression force. Also highlighted is the impact of direct compression (DC) vs. roller compaction (RC).

Tablets must be of sufficiently high tensile strength and low friability to withstand breaking, chipping, crumbling or sloughing during downstream unit operations, such as tablet coating, packaging, or transportation. Typically, higher tensile strength correlates with lower friability [36]. Sufficiently low friability means that there must be less than 1% weight loss after 100 drops of the tablets in a compendial friabilator rotating at 25 rpm [37]. Preferably, tablet weight loss should be less than 0.75%. Tablet formulations containing MCC, MCC/xCMC, and MCC/HPMC and compressed at 2.2 kN force delivered sufficient tensile strength (Figure 4) across the lactoferrin concentration range (5–45%), regardless of whether tablets were produced via direct compression or with intermediate dry granulation unit operations. Tensile strength was 0.9 MPa or greater, and friable weight loss was 0.6% or less.

Formulations containing 1:1 MCC/LAC compressed at 2.2 kN exhibited tensile strengths < 0.75 MPa. Tablet friability was correspondingly higher, with several instances of weight loss > 0.75% and one instance of friability > 1%. These observations were attributed to the high bulk density and low Hausner ratio of LAC, its broad particle distribution (as shown previously in Figure 1d SEM), and its inherent mechanism of compaction by brittle fracture [38]. MCC [39] and HPMC, however, undergo plastic deformation during compaction, which has been reported to produce stronger tablets [13], thus aligning with results from the current study.

**Figure 5:**
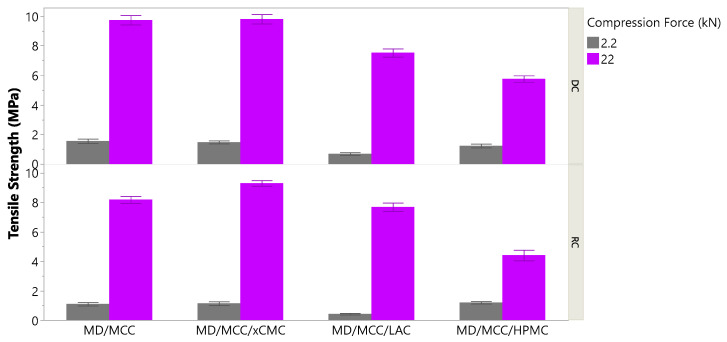
Impact of 2.2 vs. 22 kN compression force on tablet tensile strength.

Figure 5 shows that 10× greater compression force increased tensile strength drastically for tablets containing LAC, but 10× compression force drastically increased tensile strengths of all tablet formulations. Tensile strengths (5–10 MPa) observed at 22 kN compression force are considerably higher than what is necessary for sufficient tablet durability. Achieving tensile strengths above ~2 MPa is generally not necessary, in our and others’ [13,40] experiences. Since several formulations produced acceptable tablet tensile strength and friability at 2.2 kN compression force, significant time was not dedicated to optimizing either the LAC:MCC ratio or the minimal compression force necessary to bring the tablets containing LAC to sufficient strength and friability. When possible, using lower compression force extends tooling and equipment life [41], so the general rule of thumb is to compress with minimal force necessary to attain acceptable tablet physical properties.

Figure 4 also highlights that roller compacted tablets were lower in tensile strength than corresponding tablets produced via direct compression. Powders lose compactability after each densification. Powder formulations that were roller compacted, milled, and compressed to tablets were thus densified twice and consequently provided lower tensile strengths. That stated, with exception of those tablet formulations containing LAC, dry granulation rendered tablets acceptable in tensile strength and friability.

#### 3.2.2. Friability

**Figure 6:**
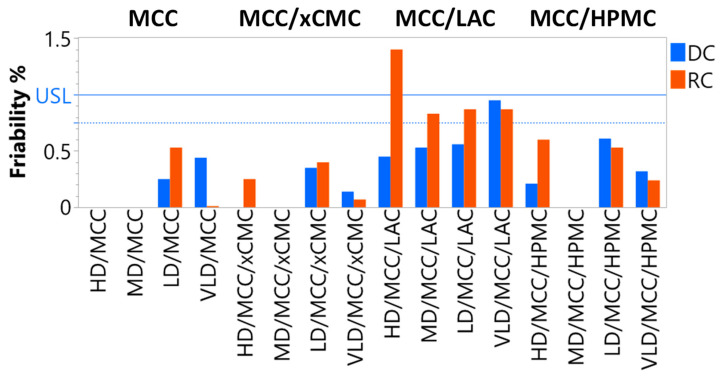
Friabilities of tablets produced via direct compression or roller compaction and tableted at 2.2 kN compression force. The solid blue reference line, USL, is the 1% friability acceptability limit, and the dashed blue line is the preferred 0.75% friability limit.

Formulations not containing LAC (i.e., MCC, MCC/xCMC, and MCC/HPMC) all had acceptably low friability of 0.6% weight loss or less (Figure 6). Tablet friability generally correlated inversely with tablet tensile strength, as expected. Consequently, tablets containing MCC/LAC were generally more friable, particularly those that were produced via dry granulation. These tablets experienced at least 0.75% friable weight loss when compressed at 2.2 kN, with one tablet formulation peaking at 1.4% weight loss. LAC-containing tablets compressed at 22 kN experienced ≤0.1% weight loss, regardless of whether the tablets were produced via direct compression or roller compaction. Again, however, lower compression force during tableting is preferred to maximize tooling and tablet press life.

#### 3.2.3. Tablet Weight Reproducibility

**Figure 7:**
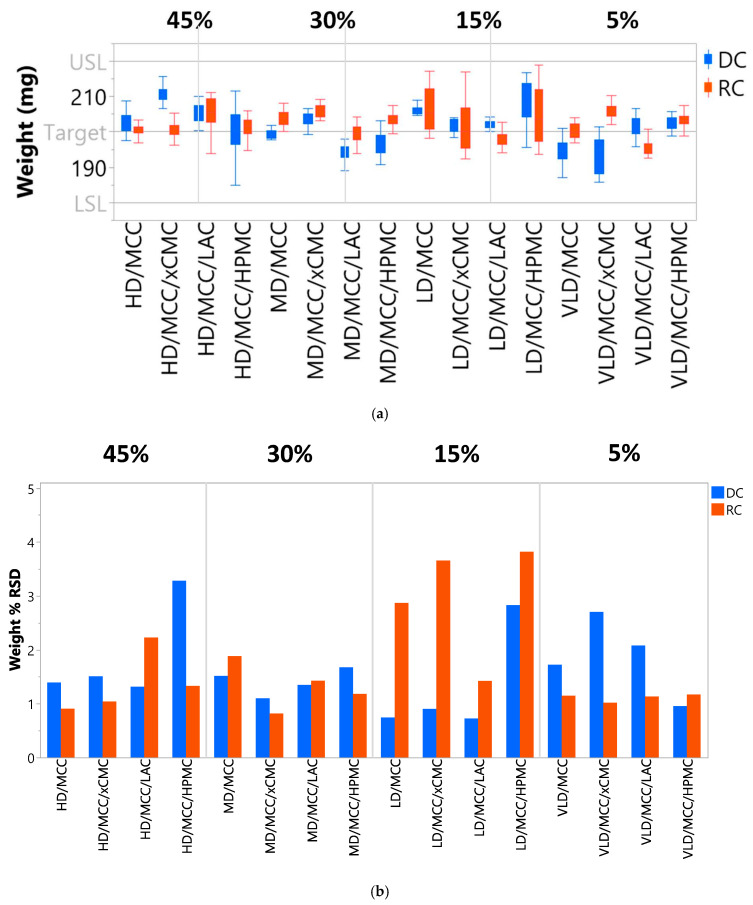
(**a**) Average weight ( $\bar{x}$ ± σ) and (**b**) weight uniformity (% RSD) of tablets produced via direct compression and roller compaction.

Inconsistent die fill during tableting is a symptom of poor flowability and can result in greater tablet weight variability and/or below-target weight. Tablet weight should fall within 90–110% of target, which in this case was 200 ± 20 mg. If tablet weight strays outside of the aforementioned limits, API dosage in individual tablets could coincidentally stray outside of the labeled limits, which should also be within 90–110% of claimed content. Too much API in the dosage form could be toxic, and not enough API could be insufficient to achieve a therapeutic effect. As shown in Figure 7a, each tablet formulation was within the weight specification of 200 ± 20 mg. Tablet weight RSD ≤ 5% is considered acceptable [42] and was achieved (Figure 7b) across the 5–45% lactoferrin concentration range via both direct compression and roller compaction.

#### 3.2.4. Content Uniformity

**Figure 8:**
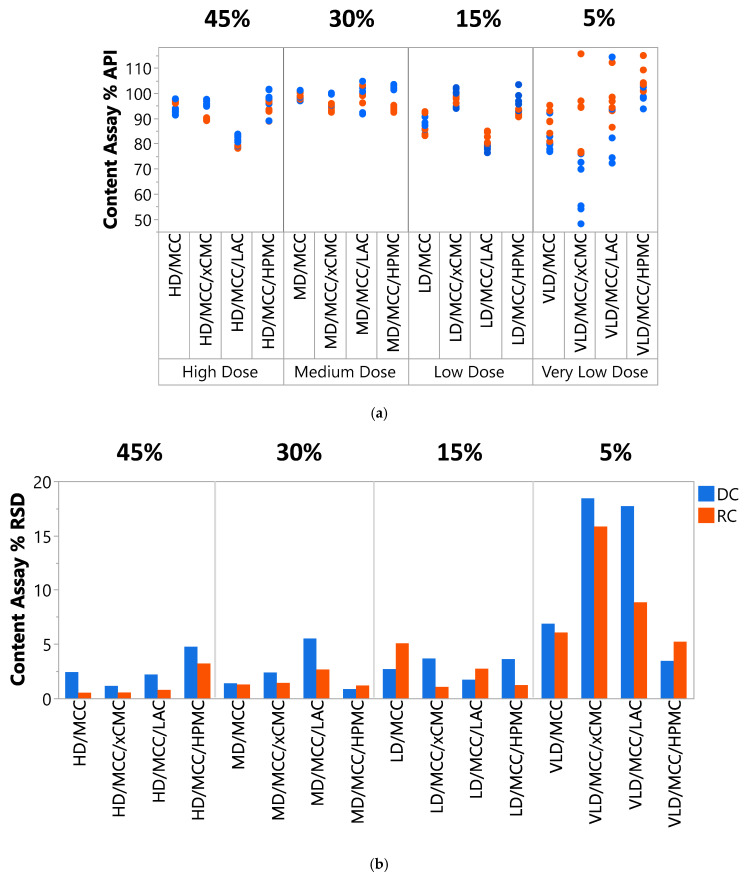
(**a**) Assayed content (%) and (**b**) content uniformity (% RSD) of the tablet formulations produced via direct compression and roller compaction. Data in blue are from direct-compressed tablets, and data in orange are from roller-compacted tablets.

Assayed lactoferrin content (%) and content uniformity (% RSD) were generally acceptable for high dose (HD, 45% lactoferrin), medium dose (MD, 30%), and low dose (LD, 15%) tablet formulations (Figure 8a,b). Content and content uniformity were unacceptable in the very low dose (VLD, 5%) tablet formulations. The authors hypothesize that lactoferrin, due to its fine particle size and very cohesive nature, did not adequately disperse throughout the powder during blending at the lowest concentration (5%), and neither the excipients used nor incorporation of intermediate roller compaction unit operations could overcome the challenges imparted when blending this very cohesive API at VLD (5% concentration). This is not surprising, considering the very cohesive appearance of lactoferrin powder (see Appendix C Figure A1). Future studies could explore blending techniques, such as trituration and geometric dilution, for improving content and content uniformity of the VLD, 5% formulations.

Inherent variability in the measurement method could also contribute to the assayed content variability observed in Figure 8a. UV assay coefficient of variation < 2% is reported [43]. This could contribute, along with actual sample content variability, to the slight scatter in the assayed content data for the 45%, 30%, and 15% lactoferrin formulations. The pronounced scatter in the VLD, 5% assayed content data, however, is clearly more attributable to actual content variability, further pointing to the need for more sophisticated blending technology at this low lactoferrin concentration.

Aside from the aforementioned challenges at the VLD 5% lactoferrin concentration, content and content uniformity generally benefited from roller compaction. Roller compacted tablets were frequently lower in % RSD (depicted in orange in Figure 8b), indicating greater content uniformity.

It should be noted that HD/MCC/LAC and LD/MCC/LAC both were measured to contain ~80% lactoferrin content (compared to 100% target; see Figure 8a). This was attributed to formulation error, in that insufficient lactoferrin may have been added during blending. Repeated testing produced similarly low measured content. Since MD/MCC/LAC was acceptable for both assayed content and content uniformity, and HD/MCC/LAC and LD/MCC/LAC had acceptable content uniformity, remaking these two LAC-containing formulations was deemed noncritical, and the data were reported as shown.

#### 3.2.5. Lactoferrin Release

**Figure 9:**
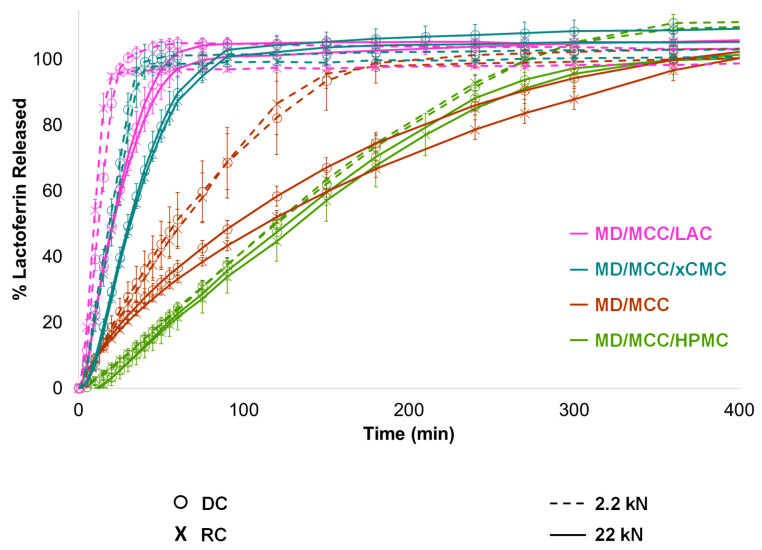
Various modes of lactoferrin release attained, depending upon excipient selection.

Only medium dose (MD; 30% lactoferrin) tablet release profiles are shown in Figure 9 for conciseness, since the HD, LD, and VLD tablet formulations released lactoferrin in corresponding order. Tablets containing 1:1 MCC/LAC provided immediate release within ~45 min; however, as stated earlier, tablets containing LAC had durability issues. The MD/MCC tablet formulation released lactoferrin over ~3 h, which was not considered immediate release. Including 2.5% xCMC with MCC provided a suitable balance of IR performance and tablet durability. Hence, the MCC/xCMC tablet formulation would be the overall preferred option for IR tablets.

The MD/MCC/HPMC tablet formulation extended release of lactoferrin over nearly 6 h, which was expected since HPMC K100LV was the excipient imparting controlled release [44]. If controlled release over a longer duration were desired, then a higher MW grade of HPMC could be incorporated.

Release performance was generally comparable from tablets produced via direct compression vs. roller compaction, with *f*_2_ similarity ranging from 53 to 95 for lactoferrin release from tablets produced at either 2.2 or 22 kN compression force. In the aforementioned case, the reference profile would have been from tablets produced at a given force via direct compression, and the test profile would have been from tablets produced at the corresponding force via roller compaction. As will be discussed in the next section, 10× compression force in most cases impacted release, the exception being controlled release HPMC matrix tablets. Controlled release performance was comparable (*f*_2_ similarity factor 64 or greater), regardless of compression force or tablet manufacturing process (direct compression vs. roller compaction). See Appendix D for more information on *f_2_* similarity factors.

It should also be noted that lactoferrin release ranged from 95–110% at the end of the testing period, as it is not common practice in our laboratory to normalize dissolution data to 100%. The 95–110% range of dissolution endpoints aligns with both (1) the content assay data in Figure 8a and (2) the previous discussion that assayed content should be within 90–110% of what is claimed.

#### 3.2.6. Impact of Compression Force

**Figure 10:**
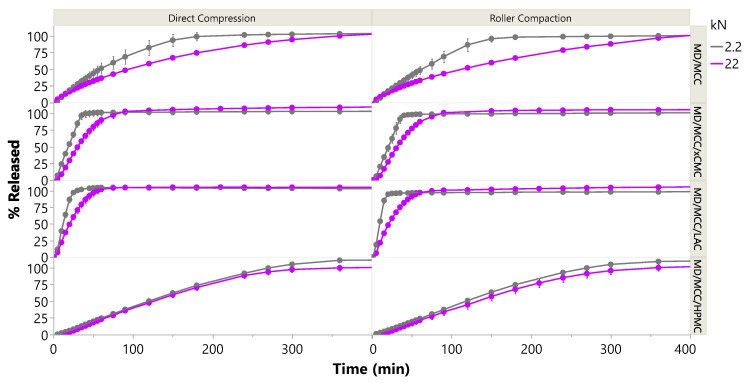
Lactoferrin release from DC and RC tablet formulations compressed at 2.2 and 22 kN.

Compression force influenced lactoferrin release and tablet disintegration with the IR formulations, particularly when only MCC was used as dry binder. This was presumably due to plastic deformation of MCC, with MCC continuing to deform under increasing compression force, increasingly entrapping lactoferrin within the plastically deformed compact. Lactoferrin release duration from the MCC-only formulation essentially doubled from 3 h to 6 h with 10× compression force, as shown in Figure 9 and Figure 10, and disintegration time tripled from 1 h to 3 h. Inclusion of xCMC at 2.5% mitigated the impact of compression force on disintegration and release, and as already discussed, did not adversely impact tensile strength or friability. With 2.5% xCMC present, the slow-down in immediate release at 10× compression force was comparable to the corresponding slow-down observed at 10× compression with tablets containing LAC.

Again, compression force exerted minimal influence on controlled release from the MD/MCC/HPMC formulation (*f*_2_ similarity factor 64 or greater). Controlled release was attained over 6 h, in alignment with the 5-h disintegration times.

#### 3.2.7. Compatibility and Stability

**Figure 11:**
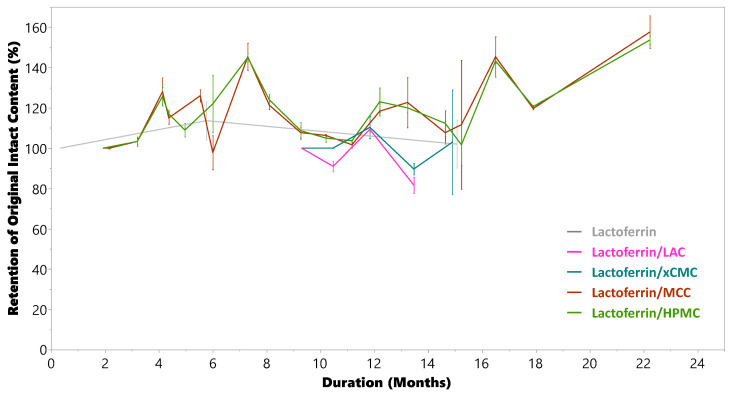
Intact lactoferrin measured vs. time from tablets containing excipients used in this study and stored at ambient conditions in the laboratory.

The data in Figure 11 come from the ELISA assay of intact lactoferrin structure measured in tablets compressed at 2.2 kN. If the structure of the lactoferrin biomacromolecule were destroyed, then it could not bind with the lactoferrin-specific antibody (supplied with the ELISA kit), thus reducing measured content of the intact biomacromolecule.

At the time of writing this manuscript, the lactoferrin has remained intact for nearly 24 months from each tablet composition stored at ambient laboratory conditions. No deleterious interactions have been observed between lactoferrin and the excipients studied. Although not shown, a single data point was gathered from tablets produced at 10× compression force as well, and there was no observed effect of 10× compression force on intact lactoferrin content.

It should be noted that noticeable ELISA measurement variability was observed, as indicated in Figure 11, along with a cyclical impact of seasonality on the measurement. The ELISA kit specifies a coefficient of variation of less than 10% for intra- or inter-assay analysis, which does not fully account for the observed variability. The authors acknowledge that this study was conducted in an ambient laboratory environment rather than using ICH controlled stability chambers. A future study should be conducted using standardized ICH stability conditions, which may also help in reducing variability in the observed measurements. That stated, there was overall no trend observed for decreasing intact lactoferrin content measured in the tablets over time.

## 4. Conclusions

Whereas previous studies focused on individual aspects related to oral formulation and delivery of biomacromolecules, such as tablet strength, protein aggregation, or bioavailability, this study followed a more comprehensive approach of investigating how excipient choice and processing operations render a cohesive biomacromolecule more processable. The results demonstrate the ability to make robust tablets across a broad API concentration range using processes that can be scaled-up for industrial production and clinical evaluation.

Tablets were successfully formulated containing lactoferrin as a very cohesive model biomacromolecule API, exhibiting acceptable tablet durability, uniformity, stability up to 24 months, and capable of delivering immediate or controlled release, depending upon excipient choice. Building on the findings of Holmfred et al. [13], who focused on compact strength and elastic recovery, the formulation principles applied in our investigation of lactoferrin as a cohesive API were similar to the principles one would consider with a cohesive small-molecule API. Our observations and results do not imply that the cohesive nature of lactoferrin is due to its high molecular weight, but rather that it is due to the physical properties of lactoferrin as a fine, cohesive powder, likely isolated via spray-drying.

An impressive outcome was the ability to produce tablets of acceptable tensile strength, friability, and performance across a broad lactoferrin concentration range of 15 to 45%, given the fine particle size and very cohesive nature of this spray-dried biomacromolecule API. At a VLD (5% lactoferrin concentration), neither excipient choice nor use of dry granulation enabled acceptable content or content uniformity. Future formulation studies at a VLD (5% lactoferrin concentration) should include powder mixing techniques, like trituration and geometric dilution.

Including LAC in the formulation did not impact lactoferrin stability or IR performance, but tablet strength and friability were unacceptable. Satisfactory strength and friability were attainable with tablets containing LAC by employing 10× greater compression force, but other excipients provided a suitable balance of tablet physical properties and IR performance at lower compression force.

If the goal is immediate release, MCC is recommended as dry binder, along with a low percentage of xCMC, to obtain a balance between tablet durability, uniformity, and IR performance. MCC, alone, is not recommended for IR tablets in this case, as MCC continues to plastically deform with increasing compression force, deleteriously impacting IR performance due to entrapment of lactoferrin in the increasingly compacted tablet. Including a low level of xCMC mitigated the impact of compression force on disintegration and release from IR tablets containing MCC.

If the goal is controlled release, matrix tablets are a robust option, with HPMC as a rate-controlling excipient. The HPMC matrix tablet formulations delivered a balance of physical properties, consistent controlled release performance, and stability, regardless of compression force or the tablet manufacturing process (direct compression or roller compaction).

Both direct compression and roller compaction were viable modes of tablet manufacture. Roller compaction did improve content uniformity, and did not adversely impact tablet physical properties or release performance, except for when LAC was included in the tablet formulations.

## Figures and Tables

**Table 1:** Materials used to produce the various tablet formulations.

Material	Name/Grade	Manufacturer/Supplier	Lot #
Bovine Lactoferrin (Lactoferrin)	95%	Parchem	B20352248
Microcrystalline Cellulose (MCC)	Avicel^®^ PH-102 NF	Roquette	P218832253
Croscarmellose Sodium (xCMC)		Spectrum Chemical	WQ0223
Lactose (LAC)	Flo Lac^®^ 100	Molkerei Meggle Wasserburg	L1015
Hypromellose (HPMC)	METHOCEL™ K100 LV	Roquette	D180K5E022
Silicon Dioxide (SiO_2_)	CAB-O-SIL^®^	CABOT	3869248
Sodium Stearyl Fumarate (SSF)	Alubra^®^ PG-100	Roquette	SF13106308

**Table 2:** Tablet formulations.

Delivery Mode	Formulation							Abbreviation
	Lactoferrin, wt.%	MCC, wt.%	xCMC, wt.%	LAC, wt.%	HPMC, wt.%	SiO_2_, wt.%	SSF, wt.%	
IR	45	54				0.5	0.5	HD/MCC
IR	30	69				0.5	0.5	MD/MCC
IR	15	84				0.5	0.5	LD/MCC
IR	5	94				0.5	0.5	VLD/MCC
IR	45	51.5	2.5			0.5	0.5	HD/MCC/xCMC
IR	30	66.5	2.5			0.5	0.5	MD/MCC/xCMC
IR	15	81.5	2.5			0.5	0.5	LD/MCC/xCMC
IR	5	91.5	2.5			0.5	0.5	VLD/MCC/xCMC
IR	45	27		27		0.5	0.5	HD/MCC/LAC
IR	30	34.5		34.5		0.5	0.5	MD/MCC/LAC
IR	15	42		42		0.5	0.5	LD/MCC/LAC
IR	5	47		47		0.5	0.5	VLD/MCC/LAC
CR	45	24			30	0.5	0.5	HD/MCC/HPMC
CR	30	39			30	0.5	0.5	MD/MCC/HPMC
CR	15	54			30	0.5	0.5	LD/MCC/HPMC
CR	5	64			30	0.5	0.5	VLD/MCC/HPMC

IR—immediate release; CR—controlled release; HD—high dose; MD—medium dose; LD—low dose; VLD—very low dose.

**Table 3:** Tableting parameters.

Tablet Press	16-Station Manesty Beta Rotary Press with Small Baffle Feeder System
Tooling	0.3125 in (7.94 mm) round concave, tooling positioned at every other station in the turret
Tablet target weight	200 mg total weight per tablet
Compression force	500 and 5000 lb (2.2 & 22 kN)
Turret speed	15 RPM

**Table 4:** Dissolution testing parameters.

**Equipment configuration**	USP II paddle method with tablet placed in suspended hanging basket
**Replicates**	6 tablets per formulation (n = 6)
**Dissolution media**	900 mL pH 7.4 phosphate buffer
**Temperature**	37 ± 0.5 °C
**Paddle speed**	100 RPM
**UV absorbance**	226 nm
**UV cell path length**	10 mm
**Tablet placement**	In suspended basket hanging 2 cm above paddle

**Table 5:** Bulk density, tapped density, and Hausner ratio for the neat excipients and for one of the high-dose IR formulations, HD/MCC (45% lactoferrin).

Material	Bulk Density (g/cc)	Tapped Density (g/cc)	Hausner Ratio
Lactoferrin	0.267 ± 0.005	0.440 ± 0.001	1.647 ± 0.030
MCC	0.320 ± 0.002	0.476 ± 0.003	1.486 ± 0.010
xCMC	0.494 ± 0.006	0.759 ± 0.001	1.536 ± 0.016
LAC	0.597 ± 0.006	0.730 ± 0.000	1.224 ± 0.012
HPMC	0.281 ± 0.001	0.505 ± 0.002	1.796 ± 0.011
HD/MCC	0.362 ± 0.002	0.541 ± 0.004	1.493 ± 0.004

**Table 6:** *D*_10_, *D*_50_, *D*_90_, and *D*[4,3] Malvern particle size attributes.

Sample Name	D_10_ (µm)	D_50_ (µm)	D_90_ (µm)	D[4,3] (µm)
Lactoferrin	4.9	15.4	30.6	16.9
MCC	42.1	127	263	174
xCMC	25.2	59.5	119	66.8
LAC	21.4	68.9	157.7	80.9
HPMC	28.8	79.1	199	99.2

## Data Availability

The original contributions and data presented in this study are included in the figures and tables of this article. Further inquiries can be directed to the corresponding author.

## Abbreviations

The following abbreviations are used in this manuscript:

API	Active Pharmaceutical Ingredient
OSDF	Oral Solid Dosage Form
MCC	Microcrystalline Cellulose
xCMC	Croscarmellose
LAC	Lactose
HPMC	Hydroxypropyl Methylcellulose
SiO_2_	Silicon Dioxide
SSF	Sodium Stearyl Fumarate
BSA	Bovine Serum Albumin
PSD	Particle Size Distribution
HD	High Dose
MD	Medium Dose
LD	Low Dose
VLD	Very Low Dose
DC	Direct Compression
RC	Roller Compaction
IR	Immediate Release
CR	Controlled Release
USP	United States Pharmacopeia
ASTM	American Society for Testing and Materials
SEM	Scanning Electron Microscopy
ELISA	Enzyme-Linked Immunosorbent Assay
min	Minutes
h	Hours
$\bar{x}$	Mean
σ	Standard Deviation
RSD	Relative Standard Deviation

## Appendix A. ELISA Methodology

Calibration standards were prepared by adding 17 mg of lactoferrin to 100 mL milliQ water in a vial, which was then capped and rolled under refrigeration (4.8 °C) for at least 2 h. This served as the lactoferrin stock solution used to make the calibration standards. Chromogen, working buffer stock, washing buffer stock, and stop solutions (all supplied with the ELISA assay kit) were removed from the storage refrigerator (6 °C) the morning prior to analysis and equilibrated to ambient room temperature. Working buffer stock was diluted 5× with milli-Q water; i.e., 16 mL milli-Q water was added to 4 mL working buffer stock. Washing buffer stock was diluted 20× prior to use; i.e., 19 mL milli-Q water was added to 1 mL washing buffer stock. Lactoferrin stock (0.1 mL) was diluted with 0.9 mL working buffer to make a 10× dilution. The 10× dilution was mixed together by pulling and expectorating from the pipette 5 times, followed by vortexing for a couple of seconds. From the 10× dilution, 0.1 mL was further diluted with 0.9 mL working buffer to make a 100× dilution. The 100× dilution was mixed together by drawing in and expectorating from the pipette 5 times, followed by vortexing for a couple of seconds. Eight 2 mL centrifuge tubes were prepared for the calibration standards and labeled 1–8. Working buffer (1 mL) was pipetted into tubes 1 and 8. Working buffer (0.400 mL) was pipetted into tubes 2 to 7. A sample of 0.050 mL of the 100× dilution was pipetted into tube 1 and mixed. A sample of 0.040 mL of the 100× dilution was pipetted into tube 8 and mixed by drawing in the pipette and expectorating 5 times, followed by vortexing for a couple of seconds. From tube 1, 0.400 mL was pipetted into tube 2, rinsing the pipette tip 5 times by drawing in and expectorating, followed by vortexing for a couple of seconds. From tube 2, 0.400 mL was pipetted into tube 3, rinsing the pipette tip 5 times by drawing in and expectorating, followed by vortexing for a couple of seconds. Subsequent serial dilutions were continued through to tube 6. Tube 7 contained only working buffer as blank solution (no lactoferrin). A sample of 0.100 mL of each calibration standard was pipetted into each well in one row of the ELISA assay plate. This row was used to generate the calibration curve.

## Appendix B. Methodology for Preparing Dissipated Tablet Samples for ELISA Analysis

Five 2 mL centrifuge tubes, tubes A to E, were prepared for each sample. More or fewer tubes were used, depending upon the concentration of lactoferrin in the tablet. Working buffer (0.9 mL) was pipetted into each tube. A sample of 0.1 mL of dissipated tablet sample was pipetted into tube A, followed by drawing in and expectorating from the pipette 5 times and then vortexing for a couple of seconds. From tube A, 0.1 mL was pipetted into tube B, followed by drawing in and expectorating from the pipette 5 times and then vortexing for a couple of seconds. From tube B, 0.1 mL was pipetted into tube C, followed by drawing in and expectorating from the pipette 5 times and then vortexing for a couple of seconds. From tube C, 0.1 mL was pipetted into tube D, followed by drawing in and expectorating from the pipette 5 times and then vortexing for a couple of seconds. From tube D, 0.1 mL was pipetted into Tube E, followed by drawing in and expectorating from the pipette 5 times and then vortexing for a couple of seconds. From tube E, 0.1 mL was pipetted into a well of the ELISA assay plate. The above steps were repeated for each of the remaining five dissipated tablet samples to make a total of n = 6 repetitions for each tablet formulation.

The assay plate was covered with microplate sealing tape (Thermofisher Scientific, Waltham, MA, USA, part number 9503130), and the plate was placed into the Tecan plate reader. The plate was shaken for 3 min and held for 27 min at 23 °C. The assay plate was removed from the Tecan instrument, and the wells were washed out 5 times by filling the wells with washing buffer and then tapping out onto paper towels to empty. For the last rinse, washing buffer was left in the wells for 2 min before tapping out onto the paper towel to empty.

The tube containing antibody (supplied with the ELISA kit) was taken out of the storage refrigerator (6 °C) and equilibrated to ambient room temperature for approximately 30 min. Note: One tube of diluted antibody solution is needed per well strip. A sample of 0.990 mL of working buffer was added into a new small vial. A sample of 0.010 mL of equilibrated antibody solution was added to the vial. A sample of 0.100 mL of diluted antibody solution was added to each of the 5× rinsed wells of the assay plate. The assay plate was again covered with microplate tape and placed into the Tecan for 3 min of shaking, followed by holding to equilibrate at 23 °C for 27 min. The assay plate was removed from the Tecan, and the wells were washed out 5 times with washing buffer. For the last rinse, washing buffer was left in the wells for 2 min before emptying. A sample of 0.100 mL of chromogen solution (equilibrated to ambient room temperature) was added to each of the 5× rinsed wells of the assay plate. The assay plate was covered with sealing tape and placed into the Tecan. The assay plate was shaken for 3 min and then held to equilibrate at 23 °C for 7 min. The assay plate was removed from the Tecan, and 0.100 mL of stop solution (supplied with the ELISA assay kit) was added to each of the wells. The assay plate was immediately placed back into the Tecan, this time without microplate tape. The plate reader was set to hold for 1 min, and the UV spectrophotometer then analyzed the wells at 450 nm wavelength. Intact lactoferrin content was determined upon completion of analysis, using the calibration standards for reference (see the previous section for calibration standard preparation).

## Appendix D. *f*_2_ Similarity Factors

**Table A1:** *f*_2_ similarity factors for release profiles from tablets produced at given compression force. DC tablet release profile was the reference, and RC tablet release profile the was test.

Delivery Mode	Abbreviation	*f*_2_, DC (Ref) vs. RC (Test) 2.2 kN	*f*_2_, DC (Ref) vs. RC (Test) 22 kN
**IR**	MD/MCC	81	67
**IR**	MD/MCC/xCMC	71	79
**IR**	MD/MCC/LAC	53	74
**CR**	MD/MCC/HPMC	95	87
